# Mechanical, Biological and In Vitro Degradation Investigation of Braided Scaffolds for Tendon and Ligament Tissue Engineering Based on Different Polycaprolactone Materials with Chitosan-Graft-PCL Surface Modification

**DOI:** 10.3390/polym16162349

**Published:** 2024-08-20

**Authors:** Caroline Emonts, Benedict Bauer, Johannes Pitts, Yvonne Roger, Andrea Hoffmann, Henning Menzel, Thomas Gries

**Affiliations:** 1 Institut für Textiltechnik, RWTH Aachen University, 52074 Aachen, Germany; 2 Institute for Technical Chemistry, Braunschweig University of Technology, 38106 Braunschweig, Germany; 3 Hannover Medical School, Department of Orthopedic Surgery, Graded Implants and Regenerative Strategies, Laboratory of Biomechanics and Biomaterials, 30625 Hannover, Germany; 4 Niedersächsisches Zentrum für Biomedizintechnik, Implantatforschung und Entwicklung (NIFE), 30625 Hannover, Germany

**Keywords:** tendon, ligament, tissue engineering, degradation, PCL grades, surface modification, braiding, macroporous scaffold, chitosan-graft-PCL, fiber cross-section

## Abstract

Injuries to tendons and ligaments are highly prevalent in the musculoskeletal system. Current treatments involve autologous transplants with limited availability and donor site morbidity. Tissue engineering offers a new approach through temporary load-bearing scaffolds. These scaffolds have to fulfill numerous requirements, the majority of which can be met using braiding combined with high-strength polycaprolactone (PCL) fibers. Considering regulatory requirements, several medical-grade PCL materials were assessed regarding their mechanical, degradational and cell biological properties. In the course of the investigation, an excellent fiber tensile strength of up to 850 MPa was achieved. The fibers were braided into multilayer scaffolds and scaled to match the human ACL. These were characterized regarding their morphology and their mechanical and degradational properties. Two strategies were followed to provide biological cues: (a) applying a chitosan-graft-PCL surface modification and (b) using non-circular fiber morphologies as topographical stimuli. Cell vitality assays showed generally positive cytocompatibility and no impairments due to the surface modification or material grade. The best cell vitality was achieved with a scaffold consisting of snowflake-shaped monofilaments combined with a 25° braiding angle. The surface modification equips the scaffold with a release platform for function molecules (as recently demonstrated) so that a holistic approach to addressing the numerous requirements is provided.

## 1. Introduction

Tendon and ligament injuries can cause significant health problems for individuals, leading to work loss and high healthcare costs [1,2]. While current treatments like biological replacements with autografts or allografts exist, they have limitations such as donor site morbidity, tissue rejection, limited tissue availability and disease transmission [3]. Synthetic prostheses have also shown drawbacks in terms of long-term mechanical durability [4]. Tissue engineering is a promising alternative that combines the short-term benefits of prosthetic devices with the avoidance of long-term deficits [5]. However, the material requirements of tissue engineering are challenging, including controllable degradation, biocompatibility, mechanical strength, maintenance during tissue regeneration, biofunctionality and processability [6,7,8]. Previous tissue engineering approaches have used either synthetic, degradable polyesters or natural polymers [5,9].

Degradable polyesters, such as polylactide acid (PLA), polyglycolide acid (PGA), and their copolymer polylactide-co-glycolide acid (PLGA), are easy to process, available in large quantities, and have good primary stability compared to natural polymers. However, excessive strength loss during degradation has been reported [5,10,11]. Additionally, the acidic degradation products of PLA and PGA have been associated with an increased inflammatory reaction, which is particularly detrimental in tissues with low vascularization, such as ligaments and tendons [12,13,14,15]. While natural polymers such as collagen are biocompatible and biofunctional, they have limitations in terms of processability, batch dependency and mechanical properties [5]. Therefore, the search for the ideal material for tissue engineering of ligamentous structures continues, with mechanical stability and adequate degradation behavior remaining major challenges [6,16,17,18]. Poly-ε-caprolactone (PCL) is a promising scaffold material due to its lack of adverse effects from acidic degradation products, FDA approval for use in degradable surgical sutures, and its application in tissue engineering of tendons, bone, and cartilage [19]. Although PCL has scarcely been studied as melt-spun fibers compared to other forms, high-oriented melt-spun PCL fibers can have excellent mechanical properties and sufficient strength retention during degradation [20,21]. Based on these fibers, textile technology can be used to fabricate high-strength, load-efficient, 3D scaffolds with adjustable and interconnected pores using established methods such as braiding or weaving [22,23]. 

The mechanical properties as well as the degradation behavior are two of the central pillars for a functional material for tendon and ligament tissue engineering [22], which both depend highly on the molecular weight of the polymer as well as the processing method used [19]. Additionally, information regarding base material properties derived from different suppliers or studies is difficult to compare since the information provided (i.e., inherent viscosity versus molecular weight) and/or characterization methods are often not identical. Especially, when excellent mechanical properties are required, such as for mechanically highly stressed structures (i.e., tendons and ligaments) [24], the mechanical potential of a specific material processed by a specific process is particularly relevant. Therefore, there is a need for a systematic comparison of PCL grades for high-load biomedical applications. This holds especially true for medical-grade materials, which are costly but necessary for the application of academic knowledge to actual medical products [19]. 

In this study, we aim to address this need by systematically comparing PCL base materials of different grades (technical, research and medical) as well as from different suppliers. For each material, a process window for melt spinning high-strength filaments is identified and the resulting mechanical potential investigated using the same setup and characterization methods (i.e., mechanical characterization and molecular weight). Three candidates are selected to be further processed into braided scaffolds. Studies on degradation behavior are subject to a complex interplay of influencing factors [25,26] and thus inherently difficult to compare. Therefore, degradation kinetics are studied in vitro for up to 36 weeks at fiber and scaffold levels. 

With mechanical properties, scalability and degradation kinetics only being parts of the equation, biological properties are also investigated [23]. In the process, the influences of technical-, research- and medical-grade materials, as well as textile process parameters, on the viability of human bone marrow-derived mesenchymal stromal cells (huBMSCs) are investigated. Additionally, the efficacy of applying a chitosan-graft-PCL surface modification (which enables the incorporation of functional molecules such as growth factors) onto the PCL fibers, and its potential effects on cell responses, are studied.

## 2. Materials and Methods

### 2.1. Fiber Fabrication

**Figure 1:**
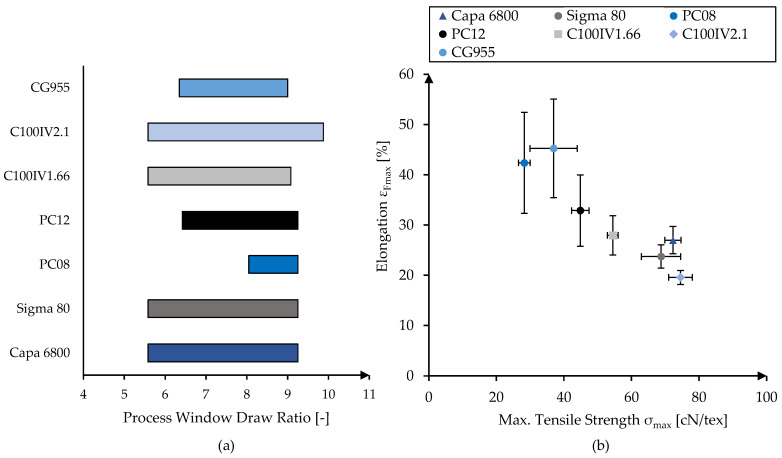
Stable draw ratio process windows of the different material grades (**a**). Resulting mechanical properties of monofilaments melt-spun at maximum draw ratio (**b**).

Different polymer pellets were acquired (see Table 1) and melt-spun into monofilaments using a single-screw extruder spinning line (Fourné Polymertechnik GmbH, Alfter, Germany). Processing temperatures were adjusted according to the melt viscosity of each material (Table 1). All materials were extruded through a circular spinneret (0.5 mm, 2 L/D), quenched in a water bath and drawn using three godet pairs before winding (SAHM 260XE, Georg Sahm GmbH & Co. KG, Eschwege, Germany), in accordance with previous studies [21]. The monofilaments were fabricated at the maximum stable draw ratio, which slightly varied between 9 and 9.88 depending on the material (Figure 1a).

Snowflake-shaped monofilaments and their circular counterparts were additionally fabricated from research-grade PCL (Sigma 80) to study the effect of fiber topography using a snowflake spinneret, as according to [21]. 

### 2.2. Braiding

The scaffolds were manufactured by using a circular braiding machine with 48 yarn carriers. The applied filament tension was 0.57 N. The one-layer scaffolds consisted of 48 filaments. The scaffolds, scaled to the mechanical properties of a human anterior cruciate ligament, consisted of 432 filaments arranged in a multilayer design. The scaffolds were made by braiding over the previous layers. All scaffolds were made with the same machine settings. The one-layer scaffolds were made from the following fibers: tg (Capa 6800), rg (Sigma 80) and mg (PC08). The nine-layer scaffolds were made from tg (Capa 6800) and rg (Sigma 80).

### 2.3. Surface Modification

To remove potential residues applied to their surface during production (e.g., during spin finishing) all samples were washed before any surface modification was applied. Briefly, 8 scaffold samples per type were placed into 45 mL centrifuge tubes and 40 mL of PBS buffer was added. The tubes were placed vertically on a laboratory shaker running at 250 rpm for 15 min at RT. Then, PBS was replaced with EtOH (aq, 70%) and washed for 1 h accordingly in order to remove possible PBS residue and microorganisms. This process was repeated two more times and subsequently the scaffolds were placed under a fume hood to evaporate EtOH for 1 h. Finally, the scaffolds were washed three times with water (Milli-Q^®^-quality) for 10 min each and dried overnight in a fume hood.

CS-g-PCL_56_ was synthesized in an improved and upscaled version of the procedure of de Cassan et al. [27] and Jing et al. [28]. The key improvements are the use of a single reaction flask instead of a separate one for chitosan dissolving, bigger and more powerful magnetic stirrers and the use of centrifugation instead of filtration during purification. This enabled 3- to 5-times the batch size per synthesis compared to Cassan et al. [27].

ε-Caprolactone was added onto a chitosan backbone via cationic ring opening polymerization. Initially, purified and dry chitosan (350 mg, 2.09 mmol, 83% DDA, Mn = 190,000–310,000, Sigma Aldrich, St. Louis, MO, USA) was dissolved in MeSO_3_H (2 mL) within a 250 mL dried Schlenk flask under a nitrogen atmosphere. The chitosan dissolved after 45 min at 50 °C. Subsequently, ε-caprolactone monomer (15.9 mL, 168.46 mmol, 72 eq.) was added to the solution and the mixture was stirred for 5 h at 50 °C. The nitrogen flow was then halted and an aqueous quench solution comprising 0.2 M KH_2_PO_4_ (43.75 mL), 10 M NaOH (7 mL) and 100 g of ice was added. The resulting crude CS-g-PCL was collected through centrifugation and subjected to vacuum drying for 48 h at room temperature. The dried CS-g-PCL was dissolved in N,N-dimethylformamide, reprecipitated in ice water, collected once more through centrifugation and vacuum dried at room temperature for an additional 48 h. The ratio X of PCL chains per glucosamine unit in CS-g-PCL was calculated from ^1^H-NMR data according to Jing et al. and noted as CS-g-PCL_X_ [28]. Dry CS-g-PCL_56_ (0.5 wt%) was dissolved in acetic acid (aq, 77 vol%) at 45 °C for 1 h. The solution (20 mL per 8 scaffolds) was transferred into a petri dish (Ø = 50 mm) and allowed to cool to RT. The scaffolds were dipped in the solution for 2 min, removed from the solution, put into dry petri dishes and placed into a vacuum oven at RT for 48 h. Then, the scaffolds were washed with water (Milli-Q^®^-quality, Merck, Darmstadt, Germany) as performed before the modification process, but this time in three intervals of 8 h each to remove cell-toxic acetic acid residue. Subsequent drying for 48 h under a fume hood finalized the process.

Modification with alginate of the CS-g-PCL_56_-scaffolds was achieved in a dipping process. The scaffolds were dipped in an alginate solution (dissolved in Milli-Q^®^-water, 5 mg/mL) for 2 min, removed and rinsed with water (Milli-Q^®^-quality). Briefly, the scaffolds were immersed in water (Milli-Q^®^-quality, 200 mL each in 8 scaffolds and a beaker) and gently stirred for 30 min at RT. The scaffolds were placed into petri dishes and dried for 48 h under a fume hood at RT.

Modification with alginate flouresceinamine (ALG-FA) was performed similarly, but with the alginate flouresceinamine solution (Milli-Q^®^-quality, 5 mg/mL) being filtered through a membrane filter by WHATMAN™ (0.20 μm, polyamide). In addition, washing with water (Milli-Q^®^-quality) was performed until the supernatant no longer emitted fluorescent light when looked at in near darkness under a handheld UV Lamp (366 nm). The water (Milli-Q^®^-quality) was changed thrice a day. The process took 5 d on a laboratory shaker (250 rpm, RT) in 45 mL centrifuge tubes filled to 40 mL. Non-modified samples also underwent this procedure in order to evaluate the necessity of CS-g-PCL modification for consecutive layer-by-layer modifications. An example is the alginate and chitosan tripolyphosphate layers carrying TGF-β_3_ growth factors introduced in a previous study by Bauer et al. on similar scaffolds [29].

### 2.4. Mechanical Characterization

Filament fineness (1 dtex = 1 g/10,000 m) was measured in accordance with DIN EN 13392 using 10 m filament segments. Maximum tensile load, tensile strength and elongation at maximum tensile load were determined in uniaxial tensile tests (STATIMAT 4U, Textechno Herbert Stein GmbH & Co. KG, Mönchengladbach, Germany) according to DIN EN 13895 [30]. For material efficiency, gauge length and testing speed were adjusted to 100 mm and 100 mm/min, respectively. The rarely occurring clamp sliders were removed. Based on DIN EN ISO 139 [31], the samples were exposed to standard textile conditions (T = 20 ± 2 °C; φ = 65 ± 4%) for at least 24 h prior to testing. 

The mechanical characterization of the braided scaffolds was performed in a uniaxial tensile test. A universal testing machine (Zmart Pro, ZwickRoell GmbH & Co. KG, Ulm, Germany) was used for this purpose. The gauge length of the specimens was 40 mm and the test was performed with a gauge length of 40 mm/min. The specimens were prepared with cardboard load application elements to prevent slippage. A clamp pressure of 10 bars and a 20 kN load cell were used for the test.

### 2.5. In Vitro Hydrolytic Degradation

Ten samples per material grade and degradation test point were prepared as described previously [21]. Deviating therefrom, the fiber lengths were increased to 250 mm in order to further reduce the risk of the clamps sliding. The period of the degradation study was set to 36 weeks in order to ensure comparability with other studies and with the ideal strength retention of 50% after 6 months of degradation formulated in the literature [32]. 

Ten samples per test point of each of the single-layer round braids (technical-grade, research-grade and medical-grade) and ten samples per test point of the multilayer round braids (technical-grade and research-grade) were stored in 1000 mL PBS at 37 °C. Each specimen had a length of 17 cm. The braids were fixed without tension to prevent the samples from contact during degradation. Only PBS resistant materials (polypropylene, stainless steel and glass) were used for the mounting to exclude any influence of degradation. 

Biweekly, the pH of the PBS was measured. In rare cases when the measured pH was not within 7.4 ± 0.2, the PBS was exchanged. The specimens were rinsed with distilled water and dried at the time of extraction. Subsequently, the specimens were prepared and tested for their tensile strength as described in Section 2.4.

### 2.6. Gel Permeation Chromatography (GPC)

A PSS SECcurity2 instrument (Mainz, Germany) equipped with a SECcurity2 vacuum degasser, a SECcurity2 TCC6500 column oven, an Agilent Infinity 1200 isocratic pump, a PSS SECcurity2 1200 refractive index detector (relative measurement against polystyrene standard) and a manual injection valve from Rheodyne were used for GPC measurements at 40 °C in THF. A triple column setup (PSS triple SDV, 1× pre-column, 2× main columns, each 10 µm particle and pore size) was installed. A concentration and flow rate of 1 g/L and 1 mL/min, respectively, were used.

### 2.7. Confocal Laser Scanning Microscopy (CLSM)

A ZEISS CLSM-510 Meta scan head connected to an Axiovert 200M (Oberkochen, Germany) was used for CLSM. Multiple scans in various depths of the CS g PCL56/alginate flouresceinamine scaffolds with the Fluar 5×/0.25 and C-Apochromat 40×/1.2 W corr. objective at 488 nm (20% laser power) excitation were carried out. For emission detection, a bandpass filter 5005-550 was used. A lambda scan series was performed to verify that the detected light was a result of fluorescence and not a reflection or from any other source. Data were processed with ZEN lite 3.7 (blue edition) by ZEISS (Oberkochen, Germany).

### 2.8. Cultivation of Bone Marrow-Derived Mesenchymal Stromal Cells

Human bone marrow-derived mesenchymal stromal cells (huBMSCs) were isolated from bone marrow as previously described [33]. Cultivation was performed in tissue culture flasks at 37 °C and 5% CO_2_ in growth medium (DMEM FG0415 from Sigma; 10% FCS from Invitrogen; 25 mM N-(2-Hydroxyethyl)piperazine-N′-2-ethanesulfonic acid (HEPES) from Roth; 100 U/mL penicillin and 100 µg/mL streptomycin from Sigma; 2 ng/mL recombinant human FGF-2 from Peprotech) until they reached a confluence of 70–80%. The cells were detached with trypsin/EDTA (Sigma) and used in the following experiments at a specific cell density.

### 2.9. Vitality Assay

Human bone marrow-derived mesenchymal stromal cells (huBMSCs) were seeded directly onto the scaffolds (20,000 cells per well of a 24-well plate) and incubated for seven days at 37 °C and 5% CO_2_. The vitality assay (Colorimetric Cell Viability Kit I (CCVK1, WST8); Promokine) was performed after 24 h, 3 and 7 days as described by the manufacturer. Briefly, the used growth medium was removed from the wells and replaced with a mixture of fresh growth media and the substrate (1/10 Vol). After 4 h of incubation at 37 °C and 5% CO_2_, 100 µL supernatant was transferred into a 96-well plate and measured in a plate reader at OD_450_. The calculations were performed with Excel.

### 2.10. Phalloidin/DAPI Staining

Parallel to the vitality assay, cells were stained with phalloidin-tetramethylrhodamine B isothiocyanate (phalloidin; cytoskeleton staining) and 4′,6-diamidino-2-phenylindole (DAPI; DNA staining) in order to analyze the morphology of the huBMSCs on the scaffolds on day 3. The cells were washed with PBS and fixated with 4% paraformaldehyde in PBS for 30 min at RT. After another two washing steps with PBS, the cells were treated with 0.1% Triton X-100 in PBS for 10 min followed by two washing steps with PBS. The cells were stained with phalloidin (0.3 µM) and DAPI (1 µg/mL) for at least 1 h at room temperature in the dark. Finally, the cells were washed twice with PBS and kept in PBS in the dark until further analysis by microscopy.

### 2.11. Statistics

The depiction of the data in the graphs is of means and standard deviation. To test for significance, a two-sided *t*-test (Excel, Microsoft Corporation, Redmond, WA, USA) was performed with significance level α = 0.05.

## 3. Results

### 3.1. Fiber Fabrication

PCL from different material grades was melt-spun with the aim of achieving maximal tensile strength, which is highly dependent on the applied draw ratio as well as the molecular weight of the material. In Figure 1a, the process window is displayed for all materials with respect to the draw ratio. Below the minimal draw ratio (natural draw ratio), the filament exhibited alternating drawn and undrawn segments. The maximal draw ratio (DR_max_) describes the draw ratio above which filament breakage occurs due to excessive tension. While Capa 6800, Sigma 80 and C100IV1.66 showed an identical process window (5.58 to 9.25), the minimal draw ratios of CG955, PC12 and PC08 are increasingly high, resulting in a narrower window of operation. DR_max_ was 9.25 for all candidates, except CG955 (DR_max_ = 9) and C100IV2.11 (DR_max_ = 9.88). 

Filaments were fabricated at each material’s DR_max_. The resulting tensile strength as well as the corresponding elongation are displayed in Figure 1b. The achieved tensile strengths varied immensely with the material used, with PC08 exhibiting the lowest (28.29 ± 1.73 cN/tex) and C100IV2.1 the highest values (74.53 ± 3.52 cN/tex). In general, with increasing tensile strength the corresponding elongation was decreased, ranging from 45.23% (CG955) and 42.36% (PC08) to 19.55% (C100IV2.1).

**Figure 2:**
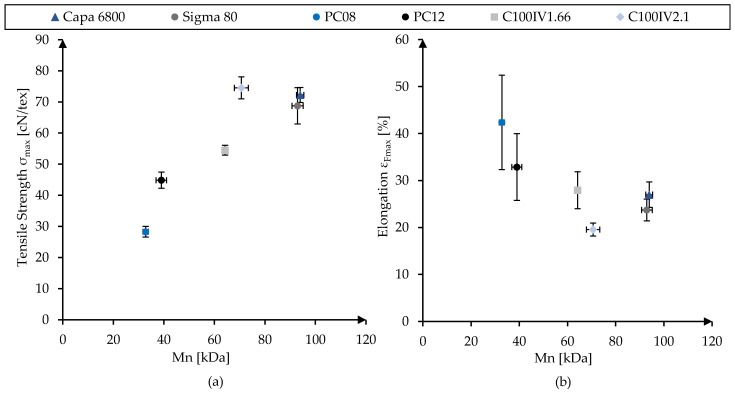
Tensile strength (**a**) and elongation at maximum tensile load (**b**) of the different polymer grades displayed as functions of their respective molecular weights M_n_.

A major influential factor for this spread is the molecular weights of the base materials, which are displayed in Figure 2. As expected, the achievable tensile strength generally increased with increasing molecular weight, with Capa 6800, Sigma 80 and C100IV2.1 located closely together at both top ends. Simultaneously, the elongation at maximum tensile load showed a declining tendency with increasing molecular weight from 42.36 ± 10.04% (PC08) to 19.55 ± 1.39% (C100IV2.1). Standard deviations decreased disproportionately with increasing molecular weight. 

Despite its molecular weight not being the highest, C100IV2.1 exhibited seemingly disproportionate values with respect to its tensile strength and respective elongation. This is attributed to the fact that its maximum stable draw ratio of 9.88 is significantly higher than the 9.25 of the other PCL grades. This in turn likely resulted in even higher orientations of the polymer chains in the axial direction.

### 3.2. In Vitro Hydrolytic Degradation of Fibers

Molecular weights and mechanical properties during hydrolytic degradation were investigated in vitro for up to 36 weeks. 

**Figure 3:**
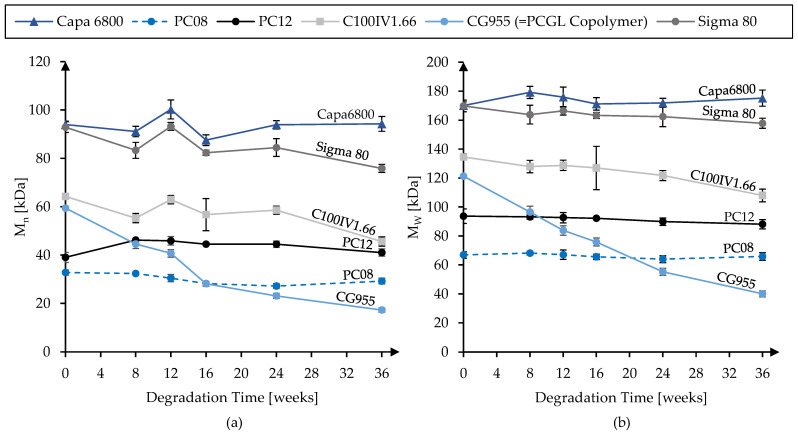
Number average molecular weight M_n_ (**a**) and weight average molecular weight M_w_ (**b**) for the different polymer grades over the course of in vitro hydrolytic degradation.

Figure 3 displays the development of the molecular weight (M_n_ and M_w_) during that timeframe. As could be expected due to the presence of PGA, the PCL-PGA-Copolymer CG955 degraded much faster than the PCL homopolymers. Its relative losses of M_n_ and M_w_ at 36 weeks compared to the initial fiber were approximately 71% and 67%, respectively. From the group of the homopolymers, C100IV1.66 and Sigma PCL 80 showed the strongest relative losses with approximately 29% and 18% for M_n_, respectively, as well as 7% and 20% for M_w_, respectively. The technical-grade PCL Capa 6800 did not exhibit a decrease in molecular weight at all.

**Figure 4:**
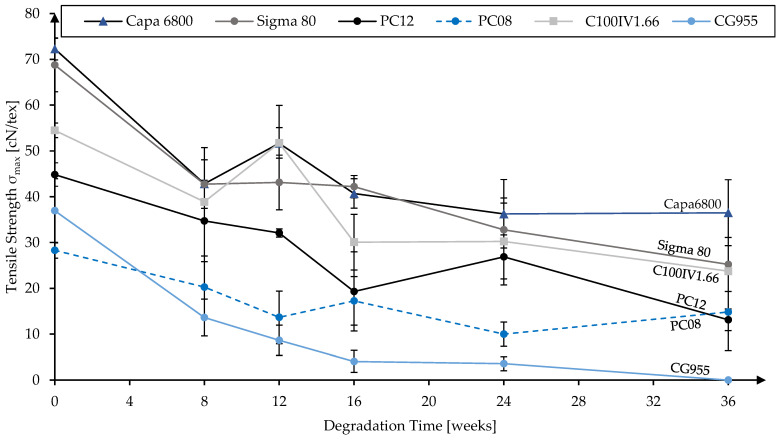
Tensile strength of monofilaments from different material grades over up to 36 weeks of in vitro hydrolytic degradation.

**Figure 5:**
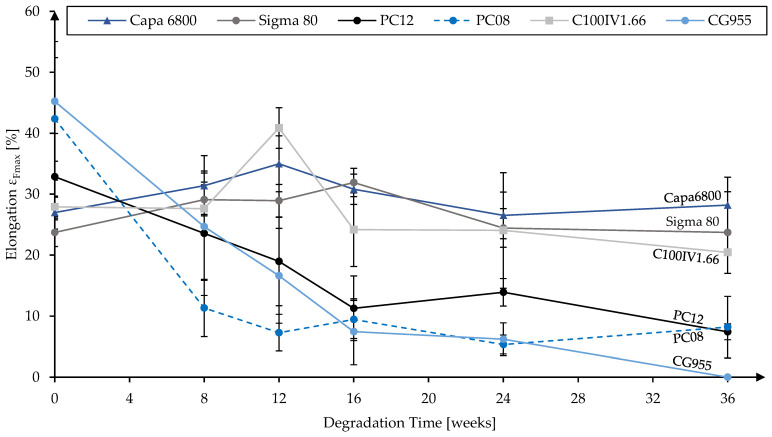
Elongation at maximum tensile load of monofilaments from different material grades over up to 36 weeks of in vitro hydrolytic degradation.

In addition to the molecular weights, mechanical properties during in vitro hydrolytic degradation were investigated. The development of tensile strength and elongation at maximum tensile load for the different material grades are presented in Figure 4 and Figure 5, respectively. 

All materials showed decreasing tensile strength over the degradation period. After 24 weeks, the strength loss compared to the non-degraded reference was by far the highest for the copolymer CG955. In this time span, its tensile strength decreased from 36.94 ± 7 cN/tex to 3.57 ± 1.53 cN/tex. From the PCL homopolymers, PC08 exhibited the most pronounced strength loss from 28.29 ± 1.73 cN/tex to 9.98 ± 2.66 cN/tex, which corresponds to ~65%. The strength loss of the other PCL grades ranged between ~40% and ~52%. After 36 weeks, the CG955 fiber samples had disintegrated and were thus untestable. From the homopolymers, PC12 showed the most pronounced strength loss from the initial 44.84 ± 2.58 cN/tex to 13.14 ± 2.36 cN/tex, which corresponds to ~71%. This is particularly interesting since its molecular weight loss was low compared to the other PCL grades. From 24 weeks to 36 weeks, Capa 6800 remained almost constant, while Sigma 80 and C100IV1.66 exhibited further decreases in tensile strength. For PC08, there was even an increase in the average tensile strength measured, which was, however, not significant (*p* = 0.117). As is not unexpected in degradation studies, the measured strength losses were not continuous for all time points.

In Figure 5, the fibers’ elongation at maximum tensile load during the degradation study are displayed. Two different modes of behavior can be observed. While one group consisting of Capa 6800, Sigma 80 and C100IV1.66 showed no significant changes after 24 weeks or 36 weeks compared to the reference, the remaining materials exhibited a certain embrittlement during degradation. Within 24 weeks, the elongations at maximum tensile load of PC08 and PC12 significantly decreased from 42.36 ± 10.04% to 5.34 ± 1.56% and from 32.86 ± 7.09% to 13.89 ± 2.26%, respectively. In the same timeframe, CG955 dropped from 45.23 ± 9.82% to 6.19 ± 2.7%. Due to the disintegration of CG955 from week 24 to week 36, the elongation at maximum tensile load was set to 0% for week 36.

### 3.3. Scaffold Fabrication

**Figure 6:**
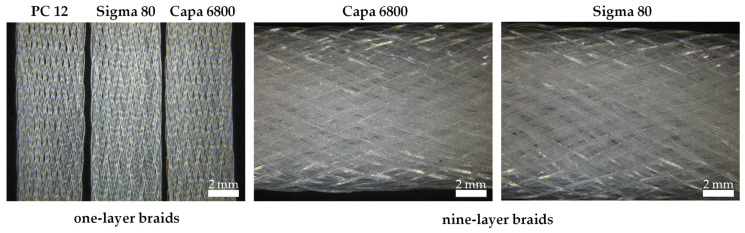
Light microscopy images of the one- and nine-layer scaffolds from the various polymer grades.

Figure 6 shows the one- and nine-layer braided scaffolds. The scaffolds made of the different polymer grades show no difference in terms of their morphological characteristics such as braiding angle and dimensions. Due to the same process parameters, it is assumed that deviations in porosity can be neglected. Table 2 lists the numbers of filaments and the resulting numbers of layers of the braided scaffolds.

#### 3.3.1. One-Layer Braids

**Figure 7:**
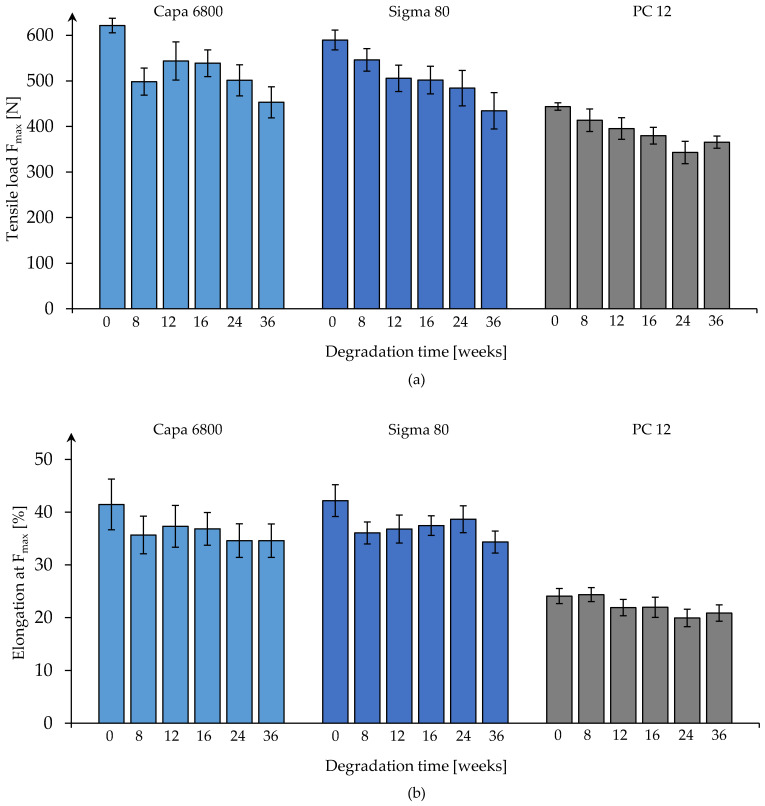
Maximal tensile load (**a**) and elongation at maximum tensile load (**b**) of one-layer braids of the different polymer grades displayed over 36 weeks degradation time.

In the case of the one-layer technical-grade (Capa 6800) braids, no steady progression of the changes in maximum tensile load during degradation was observed (Figure 7a). First, a decrease in the maximum tensile load from t0 to t8 was determined, followed by an increase from t8 to t12 (*p* < 0.05). From week 16 onwards, a significantly decreasing course was observed (*p* < 0.05).

The one-layer braids made of Sigma 80 showed a steadily decreasing progression in their maximum tensile load. A significant decrease was measured between t0 and t8 (*p* < 0.005) and between t24 and t36 (*p* < 0.05). Likewise, the one-layer braids of medical-grade PCL (PC12) showed a decreasing trend in maximum tensile load until week 24 of degradation. The decrease from t16 to t24 was significant (*p* < 0.005). The subsequent increase at t36 was not significant.

The elongations at maximum tensile load of the single-layer braids made of Capa 6800 fibers ranged from 34.5% (t36) to 37.3% (t12) (Figure 7b). There was no significant change over the course of degradation. For the braids made of Sigma 80 fibers, a significant change was only present between measurement points t24 and t36. The previous slight upward tendency was not significant. A decreasing tendency in elongation at maximum tensile load could be observed in the one-layer braids made of PC12. This decreased from 24.37% (t8) to 20.26% (t36), with significant changes from t8 to t12 (*p* < 0.005) and t16 to t24 (*p* < 0.05).

#### 3.3.2. Nine-Layer Braids

**Figure 8:**
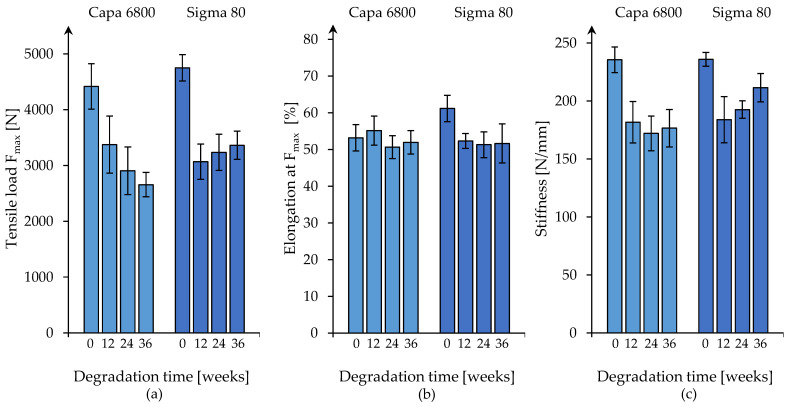
Maximal tensile load (**a**), elongation at maximum tensile load (**b**) and stiffness in the linear region (**c**) of nine-layer braids of the different polymer grades (Capa 6800 and Sigma 80) displayed over 36 weeks of degradation time.

The nine-layer round braids made of technical-grade polymer (Capa 6800) showed a continuous decrease in maximum tensile load during the degradation time (Figure 8a). Based on the maximum tensile load (4417.1 N ± 406.9 N) of the non-degraded specimens (t0), a strength retention of 60% was measured after 36 weeks (2656.8 N ± 220.1 N). A significant decrease in the maximum tensile load was measured between t0 and t12 (*p* < 0.0001), which equals a 30% decrease. 

A significant decrease in maximum tensile load of 35% was also measured after 12 weeks for the research-grade (Sigma 80) nine-layer round braids compared to the non-degraded specimens (*p* < 0.0001). A strength retention of 71% was determined after 36 weeks (3362.6 N ± 252.8 N). Between the measuring points t12, t24 and t36, no significant changes in the maximum tensile load were observed.

With regard to the application of anterior cruciate ligament replacements, the force retention of at least 50% after 6 months is considered of importance for the healing process [32]. The nine-layer braided scaffolds from both Capa 6800 and Sigma 80 fulfill this requirement. The force retention is 65% (t24 = 2905.2 N ± 426.3 N) of t0 for Capa 6800 and 68% (t24 = 2905.2 N ± 426.3 N) for Sigma 80 (t24 = 3351 N ± 232.1 N), respectively.

For the elongation at maximum tensile load, no significant change was found for the technical-grade multi-layer round braids by comparing t0 with t36 (Figure 8b). The research-grade braids showed a significant decrease (*p* < 0.0001).

The round braids made of Capa 6800 as well as of Sigma 80 showed significant decreases in stiffness in the linear range between the non-degraded samples and the first measuring point after 12 weeks (Figure 8c). Similarly, the decrease in stiffness over the course of degradation (comparing 0 and 36 weeks) was significant for the round braids from both polymer grades. For the research-grade round braids (Sigma 80), a decrease of ~10% was recorded (t0 = 235.8 N/mm ± 3.6 N/mm, t36 = 211.5 N/mm ± 13.9 N/mm). For that of the technical-grade (Capa 6800) round braid, a decrease in stiffness of ~31% was recorded (t0 = 235.5 N/mm ± 9.2 N/mm, t36 = 163.2 N/mm ± 38.1 N/mm).

### 3.4. Validation of CS-g-PCL Surface Modification

**Figure 9:**
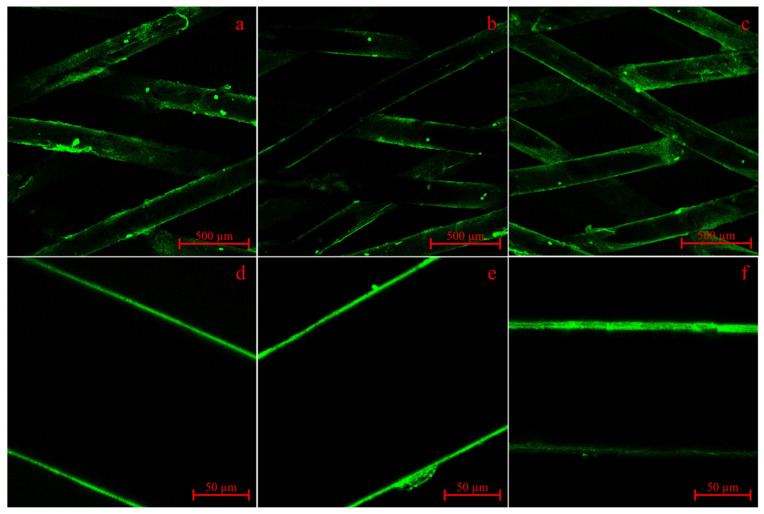
CLSM images of alginate flouresceinamine-treated CS-g-PCL_56_ scaffolds made of round monofilaments (ROMO) from research-grade PCL (Sigma 80) (**a**,**d**), technical-grade PCL (Capa 6800) (**b**,**e**) and medical-grade PCL (PC12) (**e**,**f**). Scale bars: 500 µm (**a**–**c**) and 50 µm (**d**–**f**).

As shown in Figure 9, surface modification with alginate fluoresceinamine (ALG-FA) was successful with all three types of PCL. With regards to the intensity and homogeneity of the fluorescence signal, all species performed similarly in the focal plane of the CLSM. As the samples were not perfectly flat due to their braiding structure it often looks like surface coverage might not be homogenous; however, homogenous coverage was already shown in Bauer et al. [29] via 3D scans of similar scaffolds. ALG-FA agglomerates were present even though the ALG-FA solution was filtered before the dipping process, which shows that the agglomerates should have formed after or in the modification process. In order to prove the importance of the chitosan-graft-PCL (CS-g-PCL_56_) modification for subsequent layers of alginate and chitosan TGF-β_3_ tripolyphosphate (CS-TGF-β_3_-TPP), as introduced in the previous work of Bauer et al., unmodified scaffolds of the technical and medical-grade species have been treated alike (Figure S1). These showed little to no fluorescence, indicating the necessity of the CS-g-PCL_56_ modification for alginate attachment. This has already been performed with research-grade PCL in Bauer et al. [29]

It is invaluable to thoroughly characterize new biomaterials before implantation into humans, as the material or the released compounds from the material could be toxic to cells or surrounding tissue. Endogenous primary cells are an alternative source to immortalized cell lines for analyzing the behavior of the cells on potential biomaterials, as these cells better reflect the potential response within the human body.

### 3.5. Cell Viability—PCL Grade Comparison

**Figure 10:**
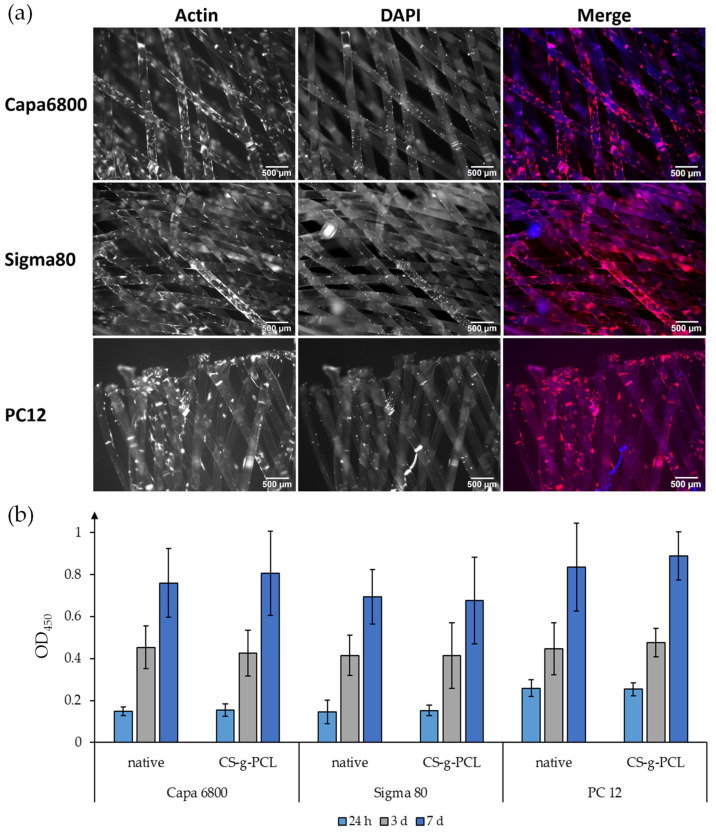
huBMSCs on textile scaffolds from different PCL grades. Shown is Capa 6800 as technical-grade, Sigma 80 as research-grade and PC12 as medical-grade PCL. The actin cytoskeleton (red; phalloidin) and the DNA (blue; DAPI) of the cells were stained and analyzed for their distribution on the scaffold as well as for their morphology after 3 days. Scale bar: 500 µm (**a**). Analysis of the vitality of the cells on materials without (native) and with CS-g-PCL coating was performed with a colorimetric cell viability kit I (WST-8; Promokine) in ten replicates for each condition after 24 h, 3 d and 7 d (**b**).

The distribution and morphology of the huBMSCs on these PCL scaffolds were analyzed by staining the actin cytoskeleton (in red, phalloidin) and the nucleus (DNA in blue, DAPI) (Figure 10a).

Cells were almost evenly distributed throughout the entire scaffold and no differences could be observed between the different grades (Figure 10a). Since phalloidin and DAPI stained the CS-g-PCL coating as well, analysis of the distribution and morphology was not performed.

The vitality assay was conducted over a time period of 7 days including the time points 24 h, 3 d and 7 d (Figure 10b). After 24 h, huBMSCs’ vitality showed a slight increase in absorbance for the medical-grade variant (PC12) in comparison to the technical (Capa 6800) and research (Sigma 80) grades. After 3 d, this trend stopped and the analysis indicated a slightly better growth of the cells on medical-grade scaffolds after 7 d, as evidenced by a slightly higher absorbance compared to the technical and research grades. The coating with CS-g-PCL did not appear to have an impact on the vitality of the cells at any of the observed time points.

### 3.6. Cell Viability—Fiber Morphology and Braiding Angle

**Figure 11:**
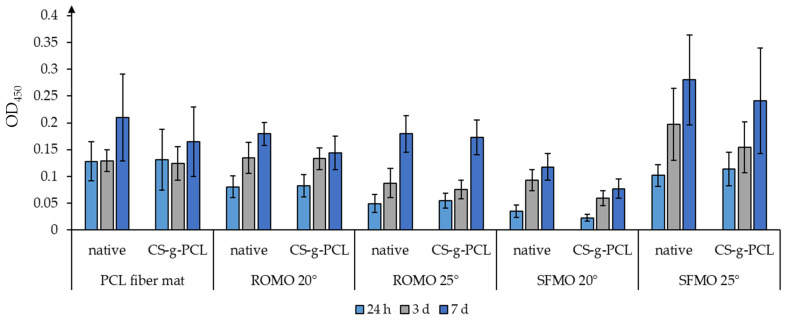
Vitality assay of huBMSCs on textile scaffolds with different fiber morphologies and braiding angles (20° and 25°). The different PCL scaffolds (PCL fiber mat, monolayer scaffolds from round monofilaments (ROMOs) and snowflake-shaped monofilaments (SFMOs)) were used without (native) or with CS-g-PCL coating. Analysis of the vitality of the cells was determined with a colorimetric cell viability kit I (WST-8; Promokine) in ten replicates for each condition after 24 h, 3 d and 7 d.

Furthermore, huBMSCs were seeded on scaffolds with different types of fiber morphology and braiding angle. The vitality of these cells was determined over a period of seven days (Figure 11).

The overall vitality increased over time for all analyzed scaffolds and the CS-g-PCL coating seemed to have a minor negative impact or even no impact on the cell vitality. The initial adherence of huBMSCs (Figure 11, shown as vitality after 24 h) varied between the different scaffolds and increased for the SFMO scaffolds with a braiding angle of 25° in comparison to ROMO 20°, ROMO 25° and SFMO 20°. Furthermore, the vitality of the cells was clearly decreased for SFMO scaffolds with a braiding angle of 20° (with and without a CS-g-PCL coating) in comparison to the same scaffold morphology with a different braiding angle (SFMO 25°) as well as for the scaffolds ROMO 20° and ROMO 25°. 

## 4. Discussion

Scaffolds for tendon and ligament replacement must meet a wide spectrum of requirements. These range from mechanical requirements, which should be similar to the native tissue, to adapted degradation kinetics and functionalization for successful in situ biologization [32,34]. High-oriented PCL is considered a promising candidate to meet these requirements but has rarely been studied to date [20]. With cross-study comparisons being inherently difficult, and considering that translation into application is facilitated by using medical-grade material, we wanted to provide a more comprehensive view on material choice. 

Therefore, different PCL base materials of different material grades, as well as from different manufacturers, were compared in this study. These were processed into fibers by melt spinning and into scaffolds by braiding. For three materials, the degradation kinetics at the fiber and scaffold levels were investigated over 36 weeks. Furthermore, the functionalizability with chitosan-graft-PCL was demonstrated and the response of huBMSCs, as one important type of regenerative cell to the different materials with and without surface modification, was analyzed. 

The choice of the PCL base material has a strong effect on the mechanical properties of the melt-spun fibers. The main influencing factor is the molecular weight. With increasing molecular weight, the maximum tensile strength increases and the elongation at maximum tensile load shows a decreasing tendency. Capa 6800 (technical-grade) and Sigma 80 (research-grade) both have the same nominal molecular weight of M_n_ = 80 kDa according to the supplier. Their process windows in terms of draw ratio are identical and the resulting mechanical properties are very similar. In view of the material costs, an early process design with the technical-grade and subsequent validation with the more expensive material can therefore be considered reasonable. The medical-grade material with the highest molecular weight was C100IV2.1, which achieved the highest draw ratio and tensile strength (74.53 cN/tex) of all the candidates, making it a promising material for the fabrication of fiber-based implants with load-bearing application. Considering that the tensile strength of comparable degradable materials in use as tendon or ligament scaffolds generally ranges between 30 and 50 cN/tex [8,35,36,37], the superior tensile strength can either be utilized to fabricate scaffolds with higher initial tensile strength or with higher porosity to facilitate cell ingrowth by using less material. 

The combination with copolymers like CG955 (PCL:PGA (95:5)) shows the adjustability of the degradation kinetics. The processability to fibers was shown and the textile processability can also be assumed based on the mechanical properties of the fibers. Therefore, it is possible to adapt the degradation profiles of scaffolds to match different applications. Comparing the degradation profiles of the fibers and braids, parallel degradation profiles were observed, especially for Capa 6800. The fibers showed the most pronounced decrease in tensile strength between week 0 and week 8. In the same timeframe, a comparable relative decrease in tensile strength could also be observed in the single-layer braids, as well as in the nine-layer braids at 12 weeks degradation time.

Scaffolds for tendon and ligament replacement are required to have a degradation profile adapted to the slow healing of the native tissue. Therefore, Vieira et al. defined the requirement that a force retention of at least 50% must be ensured after 6 months of degradation [32]. In line with this, at the fiber level, a force retention of 40–52% was measured for the PCL homopolymers after 24 weeks. For the multilayer braids, the force retention was 65% (Capa 6800)–68% (Sigma 80) after 24 weeks.

Long-term degradation studies with PCL scaffolds were also performed by Lam et al. and Leroux et al. [25,26]. Since both the scaffolds and the processing of the polymer materials differed, comparability is only possible qualitatively. Lam et al. also used research-grade PCL with an identical molecular weight of M_n_ = 80 kDa and the supplier Sigma Aldrich. Evaluation of this in vitro degradation study using 3D-printed PCL scaffolds via compression testing showed an increase in stiffness of ~150% and maximum force after three months of degradation. Both characteristic values remained unchanged during the further course of degradation. In the case of the braids made of research-grade PCL, a slight upward trend was also observed after the stiffness and maximum tensile force decreased from the start of the measurement to the degradation time of 12 weeks. 

Leroux et al. investigated the in vitro hydrolysis of PCL fiber bundles for up to 120 weeks [26]. The polymer used had a molecular weight of M_n_ = 60 kDa. The PCL fiber bundles were characterized by tensile tests. For tensile strength, a continuous decrease was measured up to 12 weeks of the degradation period. In the range of 24 weeks degradation time, an increase to about 90% of the initial value was recorded, with a subsequent decrease in tensile strength until week 120. For the Young’s modulus, also after an initial decrease, an increase (at 12 weeks) and a steady trend until 48 weeks degradation time were observed. 

The multilayer braided scaffolds presented in this study match the mechanical properties of the native anterior cruciate ligament of a young patient (<40 years). Due to the high incidence of cruciate ligament tears in the 20–25 age group [38] and the required healing capacity of the tissue for an in situ tissue engineering approach, this will be used as a requirement profile. The required maximum tensile force of a native cruciate ligament of 2160 N [39,40] is exceeded by the multilayer braided scaffolds. The stiffness, which is a crucial factor for the mobility of the knee joint, is within the range of the native tissue requirement for the scaffolds. The braided scaffolds made of all three PCL polymer grades show very good scalability in terms of maximum tensile load. The single-layer braids have a maximum tensile load of approximately 590–620 N and the nine-layer braids of 4400–4750 N. Other studies with multilayer circular braids showed the same effect of increasing the layers, resulting in an increase in maximum tensile force and stiffness [41]. The strength of the approach of using braided scaffolds for tissue engineering of the anterior cruciate ligament is that the biomimetic structure of the native ligament can be mimicked. In addition, in contrast to other textile methods such as weaving or embroidery, all fibers are positioned in the load direction so that no unnecessary synthetic material is introduced into the body. 

Several studies have dealt with comparable long-term degradable materials and textile scaffold structures. Hahn et al. investigated embroidered scaffolds made of PLA and P(LA-CL). These were investigated by means of hydrolytic and in vitro degradation. The results reached values for ultimate tensile load of 364 N, ultimate tensile elongation of 32% and stiffness of 175 N/mm, suitable for the animal model. Scaling to human application needs to be demonstrated in further studies [42]. Laurent et al. investigated multilayer circular braids made of PLCL fibers. In terms of stiffness, the braids produced were in the range of 57.9–323.7 N/mm, covering the range of the native ACL. However, the required maximum tensile force in relation to a human ACL was not achieved (37.9–323.7 N) [41]. The simultaneous achievement of stiffness, which is important for the natural kinematics of the knee, and maximum tensile strength is crucial and challenging. This challenge can be overcome with the PCL-based multilayer braids investigated in this study over the course of degradation. Mengsteab et al. have used a 3D braiding technique and PLLA fibers to produce ACL scaffolds. The maximum tensile forces were between 616.4 N and 676.2 N. Thus, the scaffolds were also below the requirement of the native ligament. However, due to the diameter being smaller than the native ligament, the possibility of further upscaling is mentioned [43]. 

As previously shown in Bauer et al. [29] CS-g-PCL_56_ modification of yarn-based fibers made from research-grade PCL was successfully verified via modification with alginate fluorescein amine (ALG-FA) and CLSM analysis. This well-established modification by de Cassan et al. has already shown the importance of the induced crystallization of CS-g-PCL on the surface of PCL scaffolds for further modification with alginate and nanoparticulate release systems like CS-TGF-β_3_-TPP [27,44]. The successful application of this surface modification on different PCL grade materials opens new possibilities. The use of medical-grade PCL, for example, allows for the fabrication of medical-accredited implant prototypes, as the main material is already approved for medical use. However, several questions must be answered beforehand. For example, the amount of growth factors that can be released from the substrates is only known for research-grade substrates. The intensity in CLSM measurements indicates that there should not be a huge difference across technical-, research- and medical-grade materials, but CLSM measurements are not suitable for precise quantitative evaluation. Only a dedicated release study analogous to Bauer et al. [29] could validate the indications shown in CLSM.

In addition to surface modifications, scaffold morphology and topography are of critical importance for cell response. Here, we used huBMSCs to investigate surface modification as well as braiding angle and fiber morphology in the context of cell proliferation and viability in in vitro cell culture. We were able to show proliferation on each individual scaffold, which is displayed via the vitality assay (WST8), as well as cell adhesion to the scaffold, demonstrated by staining of the actin cytoskeleton. The grade of PCL seems to have no impact on the vitality of the cells. For early-stage research, technical-grade material presents a cost-efficient alternative, especially in material-intensive technologies (e.g., melt spinning). The choice of medical-grade material would primarily improve the prospects of economic utilization from a regulatory perspective. Moreover, it has to be taken into consideration that vitality was determined by using one selected type of primary cell. However, further investigations are required; firstly, for a generalized assessment, and secondly, with additional cell types. This particularly may include immune cells like monocytes/macrophages, which are known to be more sensitive for additional aspects of materials than differentiation/regeneration, which is the primary approach with stem cells. Furthermore, the fiber morphology and the braiding angle, as well as the coating with CS-g-PCL, had just a slight influence on the vitality of MSCs. These observations complement previous results with PCL fiber mats (native and coated with CS-g-PCL; de Cassan et al. [44]) and seem to confirm the findings from the literature, as MSCs adhered to and survived on PCL scaffolds [45], and fibroblasts were successfully cultivated on braided as well as braided–twisted PLA scaffolds [46,47,48]. Interestingly, some of these groups and recent analyses studied cell vitality for a longer period, as well as determining the formation of extracellular matrices and gene expression during the cultivation. Therefore, the next step would be to examine this as well as to load the scaffolds with nanoparticles with growth factors and check the differentiation potential of MSCs on these scaffolds. Another approach could be a co-culturing of MSCs with other cell types, like ligamentocytes [49], in the context of the anterior cruciate ligament or another load-bearing, slow-healing tissues in the body.

## 5. Conclusions

Tissue engineering of tendons and ligaments offers a promising route to circumventing the deficiencies of current therapies yet poses multifaceted demands on the scaffolds used. In this study, a holistic approach is attempted in order to meet these demands. Mechanical and degradation-related aspects are addressed using braided, high-strength, 3D, porous scaffolds made from FDA-approved polycaprolactone, while biological cues are provided using (a) modified fiber topography and (b) a chitosan-graft-PCL surface modification, which also allows for the release of functional groups (e.g., growth factors) as shown in our recent work [29]. 

Highly oriented PCL fibers are fabricated using different grades and suppliers and characterized mechanically and chemically during in vitro degradation for up to 36 weeks. In the course of this, medical-grade PCL fibers with—to our knowledge—as yet unprecedented mechanical properties were achieved, revealing a pronounced influence of the polymer’s molecular weight in contrast to the polymer grade (technical, research or medical) the effect of which was negligible. The strength retention during degradation was also highly affected by the molecular weight and demonstrated promising values for the PCL materials with high molecular weights. These fibers were further processed into 3D porous scaffolds which were fabricated using (a) 48 fibers and (b) 432 filaments, demonstrating the scalability of textile braiding processes to match the mechanical and morphological properties of the human anterior cruciate ligament. 

The feasibility of applying a chitosan-graft-PCL surface modification was successfully demonstrated for different polymer grades and fiber topographies. Cell vitality assays showed a generally good cytocompatibility during the culture duration, while no pronounced differences between the polymer grades or between surface-modified and unmodified samples were observed. Using the chitosan-graft-PCL surface modification, the recently demonstrated attachment of a surface functionalization with clinically relevant amounts of TGF-beta equips the scaffold with another biological cue [50]. Studying its effect on cell proliferation and differentiation is amongst the next steps.

## Figures and Tables

**Table 1:** Polymer material used for melt spinning. Molecular weight (M_n_) and intrinsic viscosity (IV) are provided based on data sheets.

No.	Material	Abbreviation	Supplier	Grade	Material Properties	Processing Temperature T_P_
1	Capa^®^ 6800	Capa 6800	Ingevity Corp. (O’Hear, NC, USA)	Technical	M_n_ = 80 kDa	170–222 °C
2	PCL	Sigma 80	Merck KGaA, (Darmstadt, Germany)	Research	M_n_ = 80 kDa	170–222 °C
3	PURASORB^®^ PC08	PC08	Corbion (Amsterdam, The Netherlands)	Medical	IV = 0.8 dL/g	75–90 °C
4	PURASORB^®^ PC12	PC12			IV = 1.2 dL/g	95–125 °C
5	Capromaxx^®^ C100 (IV1.66)	C100IV1.66	Bezwada Biomedical, LLC (Hillsborough, NJ, USA)	Medical	IV = 1.66 dL/g	170–222 °C
6	Capromaxx^®^ C100 (IV2.1)	C100IV2.1			IV = 2.1 dL/g	170–222 °C
7	Capromaxx^®^ CG955	CG955			Copolymer 95:5 (PCL—PGA), IV = 1.4 dL/g	135–195 °C

**Table 2:** Material, number of filaments and the resulting number of layers of the braided scaffolds.

Scaffold	Material	Number of Filaments	#Layers
One-layer braids	Capa 6800	48	1
	Sigma 80	48	1
	PC12	48	1
Nine-layer braids	Capa 6800	432	9
	Sigma 80	432	9

## Data Availability

Data is contained within the article and Supplementary Materials.

## Supplementary Materials

The following supporting information can be downloaded at https://doi.org/10.5281/zenodo.13341195, Figure S1: CLSM images of alginate flouresceinamine-treated ROMO scaffolds made out of technical-grade PCL (a) and medical-grade PCL (b).

## References

[1] Nau T., Teuschl A. (2015). Regeneration of the anterior cruciate ligament: Current strategies in tissue engineering. World J. Orthop..

[2] Yang G., Rothrauff B.B., Tuan R.S. (2013). Tendon and ligament regeneration and repair: Clinical relevance and developmental paradigm. Birth Defects Res. C Embryo Today.

[3] Lim W.L., Liau L.L., Ng M.H., Chowdhury S.R., Law J.X. (2019). Current Progress in Tendon and Ligament Tissue Engineering. Tissue Eng. Regen. Med..

[4] Guidoin M.-F., Marois Y., Bejui J., Poddevin N., King M.W., Guidoin R. (2000). Analysis of retrieved polymer fiber based replacements for the ACL. Biomaterials.

[5] Ge Z., Yang F., Goh J.C.H., Ramakrishna S., Lee E.H. (2006). Biomaterials and scaffolds for ligament tissue engineering. J. Biomed. Mater. Res. A.

[6] Schlenker H.-J. (2006). Tissue Engineering von Bandersatz: Einfluss Mechanischer Reize auf Humane Mesenchymale Progenitorzellen und Humane Kreuzbandzellen. Doctoral Dissertation.

[7] Vunjak-Novakovic G., Altman G., Horan R., Kaplan D.L. (2004). Tissue engineering of ligaments. Annu. Rev. Biomed. Eng..

[8] Cooper J.A., Lu H.H., Ko F.K., Freeman J.W., Laurencin C.T. (2005). Fiber-based tissue-engineered scaffold for ligament replacement: Design considerations and in vitro evaluation. Biomaterials.

[9] Leong N.L., Petrigliano F.A., McAllister D.R. (2014). Current tissue engineering strategies in anterior cruciate ligament reconstruction. J. Biomed. Mater. Res. A.

[10] Sahoo S., Ouyang H., Goh J.C.-H., Tay T.E., Toh S.L. (2006). Characterization of a Novel Polymeric Scaffold for Potential Application in Tendon/Ligament Tissue Engineering. Tissue Eng..

[11] Fuoco T., Mathisen T., Finne-Wistrand A. (2019). Minimizing the time gap between service lifetime and complete resorption of degradable melt-spun multifilament fibers. Polym. Degrad. Stab..

[12] Zhang X., Wu Y., Pan Z., Sun H., Wang J., Yu D., Zhu S., Dai J., Chen Y., Tian N. (2016). The effects of lactate and acid on articular chondrocytes function: Implications for polymeric cartilage scaffold design. Acta Biomater..

[13] Gögele C., Hahn J., Elschner C., Breier A., Schröpfer M., Prade I., Meyer M., Schulze-Tanzil G. (2020). Enhanced Growth of Lapine Anterior Cruciate Ligament-Derived Fibroblasts on Scaffolds Embroidered from Poly(l-lactide-co-ε-caprolactone) and Polylactic Acid Threads Functionalized by Fluorination and Hexamethylene Diisocyanate Cross-Linked Collagen Foams. Int. J. Mol. Sci..

[14] Hutmacher D.W. (2000). Scaffolds in tissue engineering bone and cartilage. Biomaterials.

[15] Liu X., Laurent C., Du Q., Targa L., Cauchois G., Chen Y., Wang X., de Isla N. (2018). Mesenchymal stem cell interacted with PLCL braided scaffold coated with poly-l-lysine/hyaluronic acid for ligament tissue engineering. J. Biomed. Mater. Res. A.

[16] Zhang P., Han F., Chen T., Wu Z., Chen S. (2020). “Swiss roll”-like bioactive hybrid scaffolds for promoting bone tissue ingrowth and tendon-bone healing after anterior cruciate ligament reconstruction. Biomater. Sci..

[17] Petrigliano F.A., McAllister D.R., Wu B.M. (2006). Tissue engineering for anterior cruciate ligament reconstruction: A review of current strategies. Arthroscopy.

[18] Silva M., Ferreira F.N., Alves N.M., Paiva M.C. (2020). Biodegradable polymer nanocomposites for ligament/tendon tissue engineering. J. Nanobiotechnol..

[19] Woodruff M.A., Hutmacher D.W. (2010). The return of a forgotten polymer—Polycaprolactone in the 21st century. Prog. Polym. Sci..

[20] Pal J., Kankariya N., Sanwaria S., Nandan B., Srivastava R.K. (2013). Control on molecular weight reduction of poly(ε-caprolactone) during melt spinning—A way to produce high strength biodegradable fibers. Mater. Sci. Eng. C Mater. Biol. Appl..

[21] Bauer B., Emonts C., Bonten L., Annan R., Merkord F., Vad T., Idrissi A., Gries T., Blaeser A. (2022). Melt-Spun, Cross-Section Modified Polycaprolactone Fibers for Use in Tendon and Ligament Tissue Engineering. Fibers.

[22] Laurent C.P., Ganghoffer J.-F., Babin J., Six J.-L., Wang X., Rahouadj R. (2011). Morphological characterization of a novel scaffold for anterior cruciate ligament tissue engineering. J. Biomech. Eng..

[23] Thayer P.S., Goldstein A.S. (2017). Bio-Instructive Scaffolds for Tendon/Ligament Regeneration. Bio-Instructive Scaffolds for Musculoskeletal Tissue Engineering and Regenerative Medicine.

[24] Tuzlakoglu K., Reis R.L. (2009). Biodegradable polymeric fiber structures in tissue engineering. Tissue Eng. Part B Rev..

[25] Lam C.X.F., Hutmacher D.W., Schantz J.-T., Woodruff M.A., Teoh S.H. (2009). Evaluation of polycaprolactone scaffold degradation for 6 months in vitro and in vivo. J. Biomed. Mater. Res. A.

[26] Leroux A., Ngoc Nguyen T., Rangel A., Cacciapuoti I., Duprez D., Castner D.G., Migonney V. (2020). Long-term hydrolytic degradation study of polycaprolactone films and fibers grafted with poly(sodium styrene sulfonate): Mechanism study and cell response. Biointerphases.

[27] de Cassan D., Sydow S., Schmidt N., Behrens P., Roger Y., Hoffmann A., Hoheisel A.L., Glasmacher B., Hänsch R., Menzel H. (2018). Attachment of nanoparticulate drug-release systems on poly(ε-caprolactone) nanofibers via a graftpolymer as interlayer. Colloids Surf. B Biointerfaces.

[28] Jing X., Mi H.-Y., Wang X.-C., Peng X.-F., Turng L.-S. (2015). Shish-kebab-structured poly(ε-caprolactone) nanofibers hierarchically decorated with chitosan-poly(ε-caprolactone) copolymers for bone tissue engineering. ACS Appl. Mater. Interfaces.

[29] Bauer B., Emonts C., Pitts J., Buhl E.M., Eschweiler J., Hänsch R., Betsch M., Gries T., Menzel H. (2024). Topographically and Chemically Enhanced Textile Polycaprolactone Scaffolds for Tendon and Ligament Tissue Engineering. Polymers.

[30] (2003). Textiles—Monofilaments—Determination of Tensile Properties.

[31] (2011). Textiles—Standard Atmospheres for Conditioning and Testing (ISO 139:2005 + Amd.1:2011).

[32] Vieira A.C., Guedes R.M., Marques A.T. (2009). Development of ligament tissue biodegradable devices: A review. J. Biomech..

[33] Roger Y., Burmeister L., Hamm A., Elger K., Dittrich-Breiholz O., Flörkemeier T., Hoffmann A. (2020). Heparin Anticoagulant for Human Bone Marrow Does Not Influence In Vitro Performance of Human Mesenchymal Stromal Cells. Cells.

[34] Rodrigues M.T., Reis R.L., Gomes M.E. (2013). Engineering tendon and ligament tissues: Present developments towards successful clinical products. J. Tissue Eng. Regen. Med..

[35] Hahn J., Breier A., Brünig H., Heinrich G. (2018). Long-term hydrolytic degradation study on polymer-based embroidered scaffolds for ligament tissue engineering. J. Ind. Text..

[36] Hahn J. (2020). Herstellung und Charakterisierung gestickter Trägerstrukturen auf Basis abbaubarer, polymerer Fadenmaterialien für das Tissue Engineering des vorderen Kreuzbandes. Doctoral Dissertation.

[37] Chwalek K., Dening Y., Hinüber C., Brünig H., Nitschke M., Werner C. (2016). Providing the right cues in nerve guidance conduits: Biofunctionalization versus fiber profile to facilitate oriented neuronal outgrowth. Mater. Sci. Eng. C Mater. Biol. Appl..

[38] Statistisches Bundesamt (2021). Gesundheit/Fallpauschalenbezogene Krankenhausstatistik (DRG-Statistik)/Operationen und Prozeduren der Vollstationären Patientinnen und Patienten in Krankenhäusern. https://www.statistischebibliothek.de/mir/receive/DEHeft_mods_00144855.

[39] Noyes F., Grood E. (1976). The strength of the anterior cruciate ligament in humans and Rhesus. J. Bone Jt. Surg. Am..

[40] Woo S.L., Hollis J.M., Adams D.J., Lyon R.M., Takai S. (1991). Tensile properties of the human femur-anterior cruciate ligament-tibia complex: The effects of specimen age and orientation. Am. J. Sports Med..

[41] Laurent C.P., Durville D., Mainard D., Ganghoffer J.-F., Rahouadj R. (2012). A multilayer braided scaffold for Anterior Cruciate Ligament: Mechanical modeling at the fiber scale. J. Mech. Behav. Biomed. Mater..

[42] Hahn J., Schulze-Tanzil G., Schröpfer M., Meyer M., Gögele C., Hoyer M., Spickenheuer A., Heinrich G., Breier A. (2019). Viscoelastic Behavior of Embroidered Scaffolds for ACL Tissue Engineering Made of PLA and P(LA-CL) After In Vitro Degradation. Int. J. Mol. Sci..

[43] Mengsteab P.Y., Freeman J., Barajaa M.A., Nair L.S., Laurencin C.T. (2021). Ligament Regenerative Engineering: Braiding Scalable and Tunable Bioengineered Ligaments Using a Bench-Top Braiding Machine. Regen. Eng. Transl. Med..

[44] de Cassan D., Becker A., Glasmacher B., Roger Y., Hoffmann A., Gengenbach T.R., Easton C.D., Hänsch R., Menzel H. (2020). Blending chitosan-g-poly(caprolactone) with poly(caprolactone) by electrospinning to produce functional fiber mats for tissue engineering applications. J. Appl. Polym. Sci..

[45] Gurlek A.C., Sevinc B., Bayrak E., Erisken C. (2017). Synthesis and characterization of polycaprolactone for anterior cruciate ligament regeneration. Mater. Sci. Eng. C Mater. Biol. Appl..

[46] Freeman J.W., Woods M.D., Cromer D.A., Wright L.D., Laurencin C.T. (2009). Tissue engineering of the anterior cruciate ligament: The viscoelastic behavior and cell viability of a novel braid-twist scaffold. J. Biomater. Sci. Polym. Ed..

[47] Lu Q., Simionescu A., Vyavahare N. (2005). Novel capillary channel fiber scaffolds for guided tissue engineering. Acta Biomater..

[48] Lu H.H., Cooper J.A., Manuel S., Freeman J.W., Attawia M.A., Ko F.K., Laurencin C.T. (2005). Anterior cruciate ligament regeneration using braided biodegradable scaffolds: In vitro optimization studies. Biomaterials.

[49] Gögele C., Vogt J., Hahn J., Breier A., Bernhardt R., Meyer M., Schröpfer M., Schäfer-Eckart K., Schulze-Tanzil G. (2023). Co-Culture of Mesenchymal Stem Cells and Ligamentocytes on Triphasic Embroidered Poly(L-lactide-co-ε-caprolactone) and Polylactic Acid Scaffolds for Anterior Cruciate Ligament Enthesis Tissue Engineering. Int. J. Mol. Sci..

[50] Berten-Schunk L., Roger Y., Bunjes H., Hoffmann A. (2023). Release of TGF-β3 from Surface-Modified PCL Fiber Mats Triggers a Dose-Dependent Chondrogenic Differentiation of Human Mesenchymal Stromal Cells. Pharmaceutics.

